# Supplementary material for: Long-Term Prognostic Value of Myocardial Viability by Myocardial Contrast Echocardiography in Patients after Acute Myocardial Infarction: A Systematic Review and Meta-Analysis
Source: Medicina (Kaunas). 2022 Oct 11;58(10):1429. doi: 10.3390/medicina58101429 (PMC9611281; doi:10.3390/medicina58101429)
Supplement: Supplementary file 1 [file medicina-58-01429-s001.zip › medicina-1869356-supplementary.pdf]

Table S1. Literature search strategy.

A. **Literature search strategy for EMBASE**

#	Searches	result
#1	Contrast Echocardiography.mp. [mp=title, abstract, heading word, drug trade name, original title, device manufacturer, drug manufacturer, device trade name, keyword, floating subheading word, candidate term word]	5500
#2	(heart or cardia* or myocardial).mp.	2594229
#3	((heart or cardia* or myocardial) adj3 Infarc*).mp.	419683
#4	((heart or cardia* or myocardial) adj3 ischemia).mp.	115513
#5	event*.mp.	1404367
#6	death.mp.	1245477
#7	Mortality.mp.	1461027
#8	progno*.mp.	1128005
#9	diagno*.mp.	6309898
#10	predict*.mp.	2210371
#11	#8 or #9 or #10	8359526
#12	#5 or #6 or #7	3527209
#13	#1 and #2	4836
#14	#3 or #4	494270
#15	#14 and #13	1484
#16	#11 and #12	1223581
#17	#15 and #16	230
#18	limit 17 to (human and english language)	201

mp=title, abstract, heading word, drug trade name, original title, device manufacturer, drug manufacturer, device trade name, keyword, floating subheading word, candidate term word

B. Literature search strategy for Pubmed

#	Searches	Results
#1	(("myocardial infarction"[Mesh]) OR "myocardial ischemia"[Mesh])	428,229
#2	(("myocardial infarction"[Text Word]) OR "myocardial ischemia"[Text Word])	287,401
#3	#1 OR #2	485,989
#4	“Contrast Echocardiography”[text word]	2,853
#5	"heart"[text word] or "cardia*"[text word] or "myocardial"[text word]	1,723,610
#6	#4 AND #5	2,444
#7	“event*”[text word] or "Mortality"[text word] or "death"[text word]	2,536,683
#8	“progno*”[text word] or "diagno*"[text word] or "predict*"[text word]	6,480,915
#9	#7 AND #8	854,412
#10	#3 AND #6 AND #9	126
#11	animals[MeSH Terms]	23,237,606
#12	humans[MeSH Terms]	18,561,638
#13	#11 NOT #12	4,712,846
#14	#10 NOT #13	121

Figure S1. Predictive Value and Accuracy of MCE in patients after AMI.

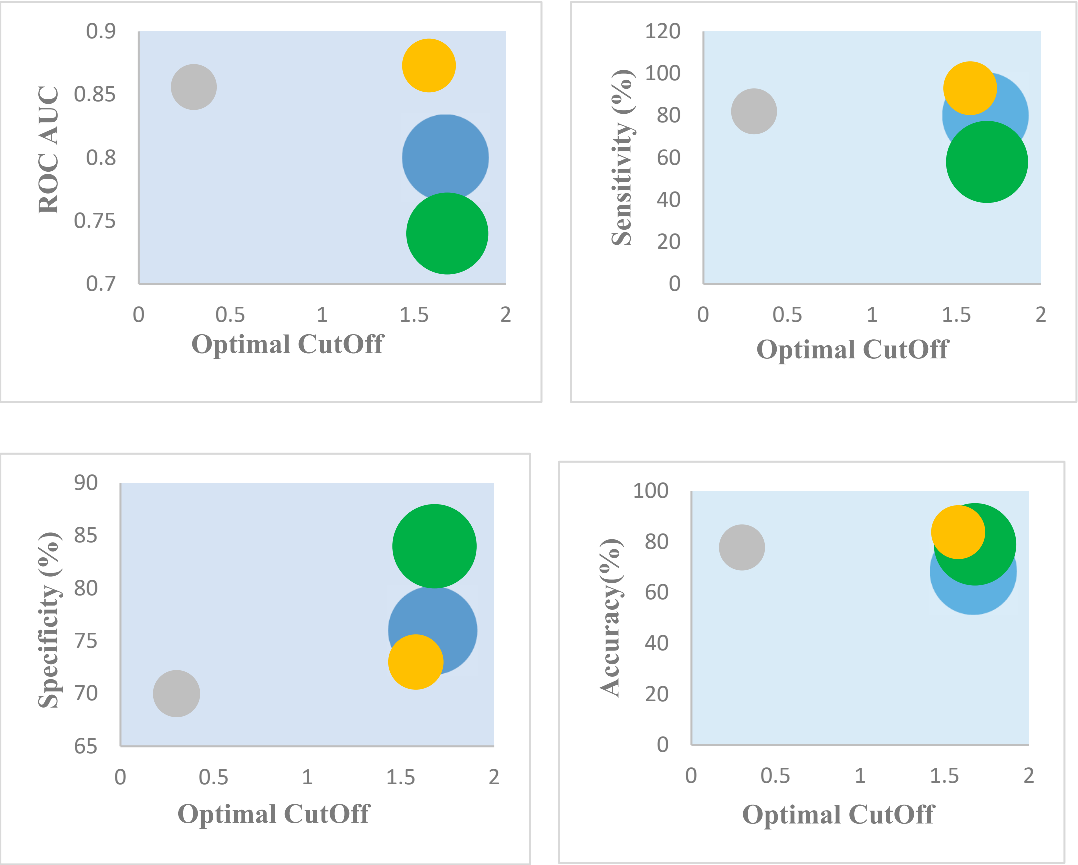

*The sizes of the bubbles indicate the number of patients. AUC: area under the curve; ROC: receiver-operating characteristic.

Publication	semiquantitative scoring system	Optimal CutOff	ROC AUC	Sensitivity (%)	Specificity (%)	Accuracy(%)
Dwivedi et al	1/2/3	1.67	0.8	80	76	68.4
Olszowska et al	1/2/3	1.68	0.74	58	84	79
Abdelmoneim et al	1/2/3	1.58	0.873	93	73	83.8
Lenz et al	**0/1/2**	**0.3**	0.856	82	70	77.8
Pooled parameters	/	/	0.84	0.80	0.78	/
Pooled 95%CI	/	/	0.80-0.87	0.64-0.90	0.69-0.85	/

Table S2. Forrest Plots for sensitivity analysis for pooled adjusted RR, performed by using a leave-one-out of the weight of each study.

A.

1 removal of the study by Abdelmoneim *et al.*

Study or Subgroup	log[Risk Ratio]	SE	Weight	Risk Ratio IV, Random, 95% CI
Abdelmoneim 2015	1.2267	0.5371	0.0%	3.41 [1.19, 9.77]
Bolognese 2004	0.8198	0.3338	24.7%	2.27 [1.18, 4.37]
Dwivedi 2007	0.2311	0.0739	42.0%	1.26 [1.09, 1.46]
Lenz 2016	1.3635	0.7165	9.6%	3.91 [0.96, 15.92]
Olszowska 2010	0.8755	0.3494	23.7%	2.40 [1.21, 4.76]
**Total (95% CI)**			**100.0%**	**1.89 [1.16, 3.10]**

Heterogeneity: Tau^2 = 0.15; Chi^2 = 8.20, df = 3 (P = 0.04); I^2 = 63%
Test for overall effect: Z = 2.53 (P = 0.01)

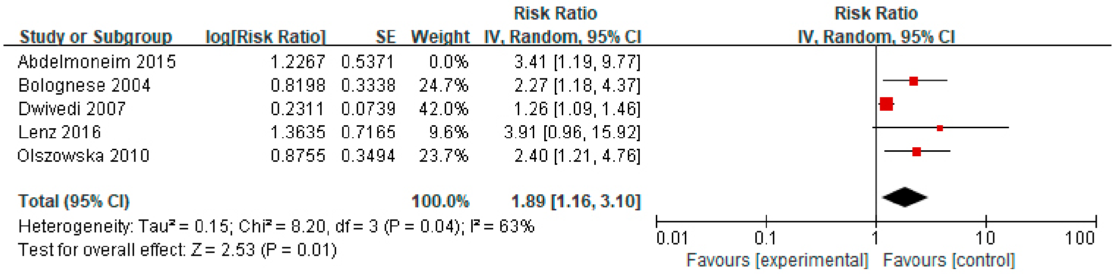

2 removal of the study by Bolognese *et al.*

Study or Subgroup	log[Risk Ratio]	SE	Weight	Risk Ratio IV, Random, 95% CI
Abdelmoneim 2015	1.2267	0.5371	18.4%	3.41 [1.19, 9.77]
Bolognese 2004	0.8198	0.3338	0.0%	2.27 [1.18, 4.37]
Dwivedi 2007	0.2311	0.0739	41.4%	1.26 [1.09, 1.46]
Lenz 2016	1.3635	0.7165	12.8%	3.91 [0.96, 15.92]
Olszowska 2010	0.8755	0.3494	27.4%	2.40 [1.21, 4.76]
**Total (95% CI)**			**100.0%**	**2.09 [1.14, 3.81]**

Heterogeneity: Tau^2 = 0.22; Chi^2 = 8.73, df = 3 (P = 0.03); I^2 = 66%
Test for overall effect: Z = 2.40 (P = 0.02)

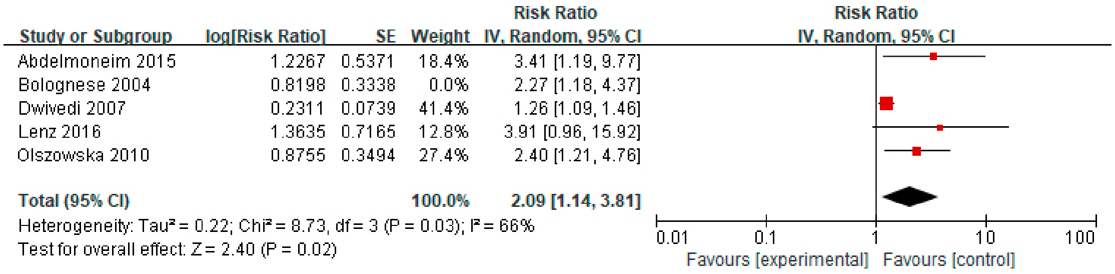

3 removal of the study by Dwivedi *et al.*

Study or Subgroup	log[Risk Ratio]	SE	Weight	Risk Ratio IV, Random, 95% CI
Abdelmoneim 2015	1.2267	0.5371	15.4%	3.41 [1.19, 9.77]
Bolognese 2004	0.8198	0.3338	39.7%	2.27 [1.18, 4.37]
Dwivedi 2007	0.2311	0.0739	0.0%	1.26 [1.09, 1.46]
Lenz 2016	1.3635	0.7165	8.6%	3.91 [0.96, 15.92]
Olszowska 2010	0.8755	0.3494	36.3%	2.40 [1.21, 4.76]
**Total (95% CI)**			**100.0%**	**2.58 [1.71, 3.90]**

Heterogeneity: Tau^2 = 0.00; Chi^2 = 0.80, df = 3 (P = 0.85); I^2 = 0%
Test for overall effect: Z = 4.51 (P < 0.00001)

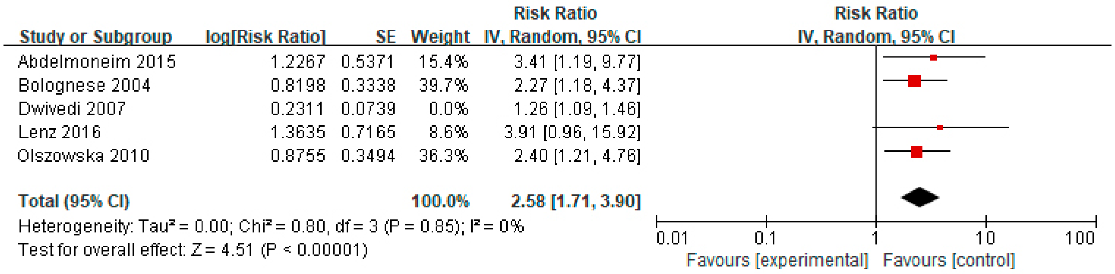

4 removal of the study by Lenz *et al.*

Study or Subgroup	log[Risk Ratio]	SE	Weight	Risk Ratio IV, Random, 95% CI
Abdelmoneim 2015	1.2267	0.5371	14.3%	3.41 [1.19, 9.77]
Bolognese 2004	0.8198	0.3338	23.7%	2.27 [1.18, 4.37]
Dwivedi 2007	0.2311	0.0739	39.3%	1.26 [1.09, 1.46]
Lenz 2016	1.3635	0.7165	0.0%	3.91 [0.96, 15.92]
Olszowska 2010	0.8755	0.3494	22.8%	2.40 [1.21, 4.76]
**Total (95% CI)**			**100.0%**	**1.93 [1.18, 3.17]**

Heterogeneity: Tau^2 = 0.16; Chi^2 = 8.98, df = 3 (P = 0.03); I^2 = 67%
Test for overall effect: Z = 2.62 (P = 0.009)

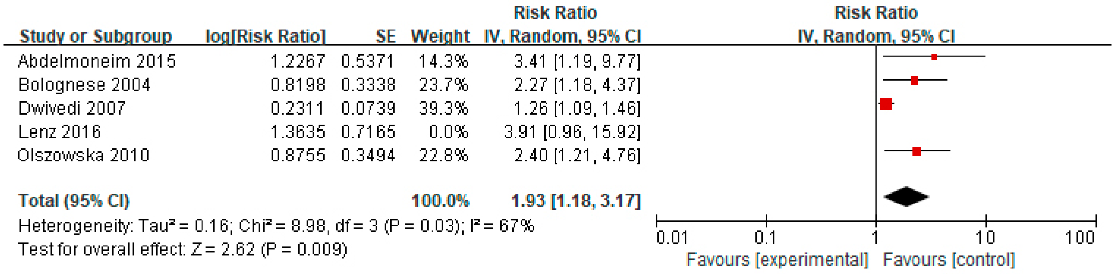

5 removal of the study by Olszowska *et al.*

Study or Subgroup	log[Risk Ratio]	SE	Weight	Risk Ratio IV, Random, 95% CI
Abdelmoneim 2015	1.2267	0.5371	17.8%	3.41 [1.19, 9.77]
Bolognese 2004	0.8198	0.3338	27.9%	2.27 [1.18, 4.37]
Dwivedi 2007	0.2311	0.0739	42.2%	1.26 [1.09, 1.46]
Lenz 2016	1.3635	0.7165	12.2%	3.91 [0.96, 15.92]
Olszowska 2010	0.8755	0.3494	0.0%	2.40 [1.21, 4.76]
**Total (95% CI)**			**100.0%**	**2.03 [1.14, 3.62]**

Heterogeneity: Tau² = 0.20; Chi² = 8.44, df = 3 (P = 0.04); I² = 64%
Test for overall effect: Z = 2.41 (P = 0.02)

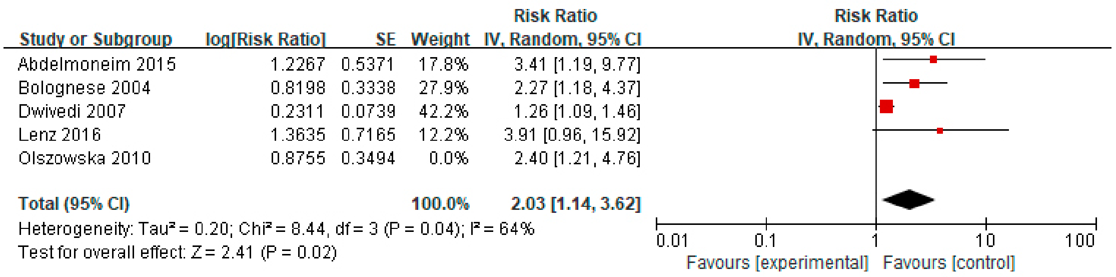

B.

1 removal of the study by Bolognese *et al.*

Study or Subgroup	log[Risk Ratio]	SE	Weight	Risk Ratio IV, Random, 95% CI
Bolognese 2004	1.3481	0.5198	0.0%	3.85 [1.39, 10.66]
Dwivedi 2007	1.5041	0.6335	36.3%	4.50 [1.30, 15.58]
Khumri 2006	0.3148	0.1214	63.7%	1.37 [1.08, 1.74]
**Total (95% CI)**			**100.0%**	**2.11 [0.69, 6.48]**

Heterogeneity: Tau² = 0.50; Chi² = 3.40, df = 1 (P = 0.07); I² = 71%
Test for overall effect: Z = 1.31 (P = 0.19)

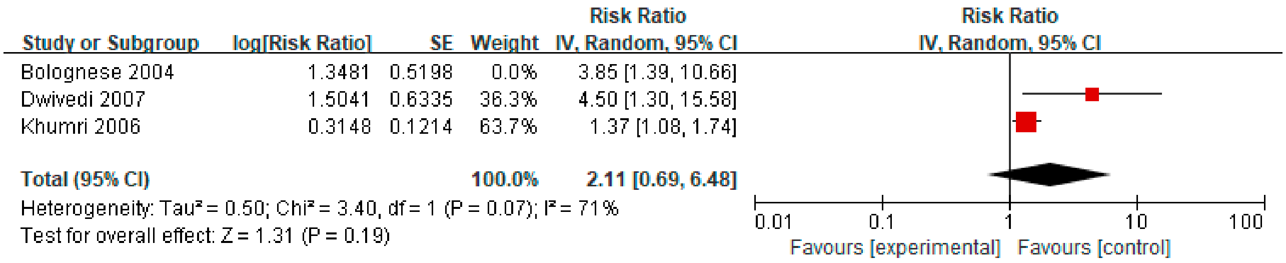

2 removal of the study by Dwivedi *et al.*

Study or Subgroup	log[Risk Ratio]	SE	Weight	Risk Ratio IV, Random, 95% CI
Bolognese 2004	1.3481	0.5198	38.0%	3.85 [1.39, 10.66]
Dwivedi 2007	1.5041	0.6335	0.0%	4.50 [1.30, 15.58]
Khumri 2006	0.3148	0.1214	62.0%	1.37 [1.08, 1.74]
**Total (95% CI)**			**100.0%**	**2.03 [0.76, 5.43]**

Heterogeneity: Tau² = 0.39; Chi² = 3.75, df = 1 (P = 0.05); I² = 73%
Test for overall effect: Z = 1.41 (P = 0.16)

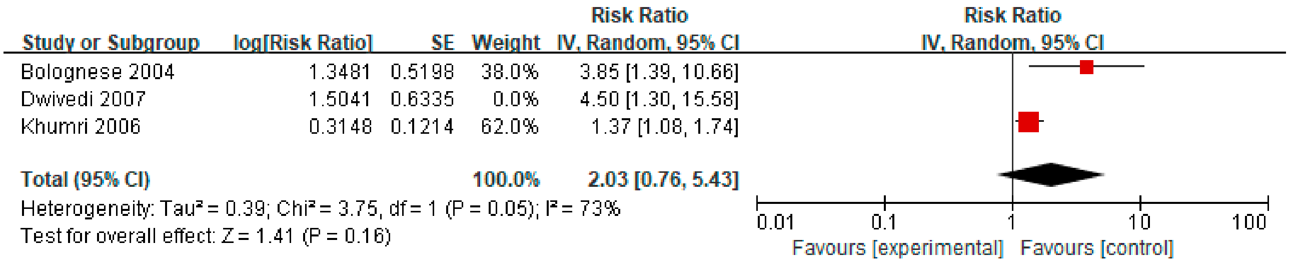

3 removal of the study by Khumri *et al.*

Study or Subgroup	log[Risk Ratio]	SE	Weight	Risk Ratio IV, Random, 95% CI
Bolognese 2004	1.3481	0.5198	59.8%	3.85 [1.39, 10.66]
Dwivedi 2007	1.5041	0.6335	40.2%	4.50 [1.30, 15.58]
Khumri 2006	0.3148	0.1214	0.0%	1.37 [1.08, 1.74]
**Total (95% CI)**			**100.0%**	**4.10 [1.87, 9.01]**

Heterogeneity: Tau² = 0.00; Chi² = 0.04, df = 1 (P = 0.85); I² = 0%
Test for overall effect: Z = 3.51 (P = 0.0004)

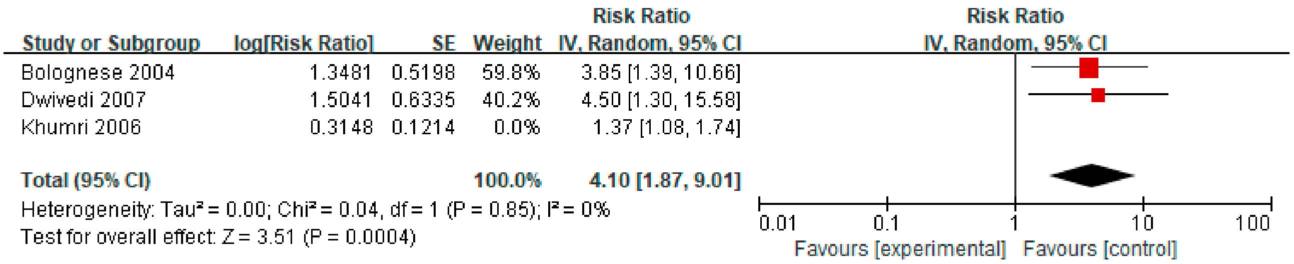

A. total cardiac events and B. hard cardiac events**.** RR = risk ratio; CI = confidence interval; SE = standard error of the hazard ratio.

Table S3. Quality Assessment.

Study	Selection				Comparability	Outcome			Quality (Total points)
	Representativeness of exposed cohort	Selection of the non-exposed cohort	Ascertainment of exposure	Outcome demonstration at start		Assessment of outcome	Follow-up long enough for outcome to occur	Adequacy of follow-up	
Bolognese L *et al.*	1	1	1	1	2	1	1	1	Good(9)
Khumri TM *et al.*	1	1	1	1	2	1	1	1	Good(9)
Dwivedi *et al*	1	1	1	1	2	1	1	1	Good(9)
Olszowska *et al.*	1	1	1	1	2	0	1	1	Good(8)
Abdelmoneim *et al.*	0	1	1	1	2	1	0	1	Good(7)
Lenz *et al.*	0	1	1	1	2	1	1	1	Good(8)

A. Newcastle-Ottawa scale for included studies. The Newcastle-Ottawa Scale (NOS) evaluates the quality of included studies based on three parts: selection, comparability and outcome. The full score for each part is 4, 2, 3, respectively, and the maximum total score is 9.

Study	Sonographers blinded to outcome	Prognostic factor defined	appropriate cut-off points	Prognostic factor measurement	Outcome described	adequacy of the analysis
Bolognese L *et al.*	YES	YES	NO	YES	YES	YES
Khumri TM *et al.*	YES	YES	NO	YES	YES	YES
Dwivedi *et al.*	YES	YES	YES	YES	YES	YES
Olszowska *et al.*	N/A	YES	YES	YES	YES	YES
Abdelmoneim *et al.*	YES	YES	YES	YES	YES	YES
Lenz *et al.*	YES	YES	YES	YES	YES	YES

B. Quality data for eligible data sets. N/A = not available
